# UDP glucuronosyltransferase 2B7 single nucleotide polymorphism (rs7439366) influences heat pain response in human volunteers after i.v. morphine infusion

**DOI:** 10.1186/cc9783

**Published:** 2011-03-11

**Authors:** KM Meissner, HM Meyer zu Schwabedissen, CG Göpfert, MD Ding, JB Blood, KF Frey, HK Kim, EK Kharasch

**Affiliations:** 1 Universitätsklinikum Greifswald, Germany; 2 Washington University in St Louis, MO, USA

## Introduction

Morphine remains the most widely used intravenous opioid in the perioperative setting worldwide. Maintaining therapeutic CNS concentrations of many opioids is confounded by considerable variability in disposition. Recent findings indicate a role for the UGT2B7 expressed in the liver, for variability of substrate effects. This phenomenon is attributed to genetic and environmental factors. However, evidence for effect variation due to UGT2B7-mediated glucuronization of morphine in humans is lacking.

## Methods

We tested the hypothesis that variations of morphine effects could be explained in part by genetic variation in the UGT2B7 gene by pupil diameter change and heat pain response in 35 healthy volunteers, who were given 0.2 mg/kg morphine i.v. over 2 hours. This abstract reports the results for the UGT2B7 (rs7439366) SNP on chromosome 4, coding for a histidine or a tyrosine at position 268, resulting in decreased enzyme activity.

## Results

**Figure 1:**
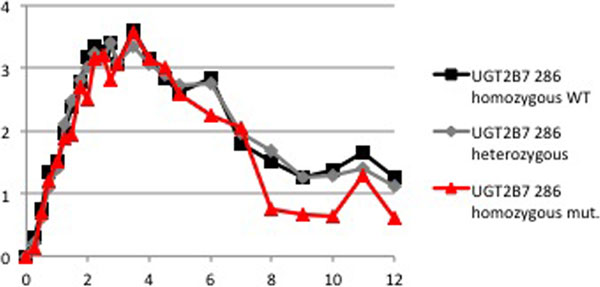
**Miosis (mm) after the start of the morphine injection**.

**Figure 2:**
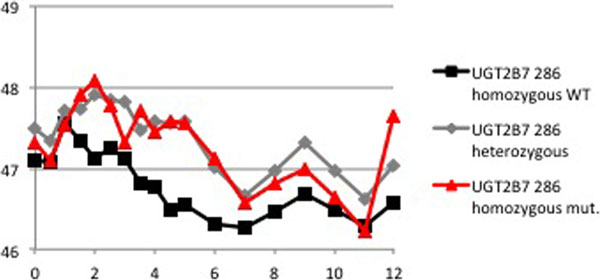
**Maximally tolerable temperatures (°C) hours after morphine injection**.

Ten subjects exhibited the wildtype, 20 were heterozygous and five were homozygous carriers of the allele. Peak effects of miosis did not differ for the three variants (Figure 1). However, while the results for heat pain response indicate almost no effect at all for wildtype subjects, carriers of the T allele experience a higher peak and an extended analgesia (Figure 2). Neither the parent drug nor the 3-glucuronide and 6-glucuronide serum concentrations differed significantly among the research subjects.

## Conclusions

While morphine effects might be influenced in part by UGT2B7 genotype, there is a differential effect on pupil contractility and heat pain response. This cannot be readily explained by drug or metabolite serum concentration and warrants further investigation, including different enzyme product effects on cerebral morphine levels.

